# A Systems-Level Analysis of Mechanisms of *Platycodon grandiflorum* Based on A Network Pharmacological Approach

**DOI:** 10.3390/molecules23112841

**Published:** 2018-11-01

**Authors:** Musun Park, Sa-Yoon Park, Hae-Jeung Lee, Chang-Eop Kim

**Affiliations:** 1 Department of Physiology, College of Korean Medicine, Gachon University, Seongnam 13120, Korea; bmusun1@gmail.com (M.P.); psy9228@gmail.com (S.-Y.P.); 2 Department of Food and Nutrition, College of BioNano Technology, Gachon University, Seongnam 13120, Korea

**Keywords:** *Platycodon grandiflorum*, Kilkyung, systems-level mechanism, network pharmacology, traditional Asian medicine

## Abstract

*Platycodon grandiflorum* (PG) is widely used in Asia for its various beneficial effects. Although many studies were conducted to understand the molecular mechanisms of PG, it is still unclear how the combinations of multiple ingredients work together to exert its therapeutic effects. The aim of the present study was to provide a comprehensive review of the systems-level mechanisms of PG by adopting network pharmacological analysis. We constructed a compound–target–disease network for PG using experimentally validated and machine-leaning-based prediction results. Each target of the network was analyzed based on previously known pharmacological activities of PG. Gene ontology analysis revealed that the majority of targets were related to cellular and metabolic processes, responses to stimuli, and biological regulation. In pathway enrichment analyses of targets, the terms related to cancer showed the most significant enrichment and formed distinct clusters. Degree matrix analysis for target–disease associations of PG suggested the therapeutic potential of PG in various cancers including hepatocellular carcinoma, gastric cancer, prostate cancer, small-cell lung cancer, and renal cell carcinoma. We expect that network pharmacological approaches will provide an understanding of the systems-level mechanisms of medicinal herbs and further develop their therapeutic potentials.

## 1. Introduction

*Platycodon grandiflorum* (PG), known as Kilkyung (in Korea), Jiegeng (in China), or Kikyo (in Japan), is widely used worldwide for its therapeutic effects on cough, phlegm, sore throat, and so on. So far, many studies focused on the biological effects of PG, such as anti-inflammatory [1,2,3], anti-cancer [4,5], anti-oxidative [6,7], and anti-obesogenic properties [8]. In particular, a number of studies investigated the efficacy of platycodin D, the main active component of PG. Platycodin D was found to have diverse pharmacological effects, such as inducing apoptosis [9,10,11,12,13], anti-obesity [14,15], and anti-inflammatory effects [16,17,18], increasing airway mucin release [19,20], and protection against hepatotoxicity [21,22]. However, PG contains various ingredients in addition to platycodin D, and many of the ingredients may work together to exert the therapeutic effects of PG.

Despite many studies trying to understand the molecular mechanisms of PG, it is still unclear how the combinations of multiple ingredients work together to exert its therapeutic effects. Since most diseases are caused by an interplay of multiple molecular components [23], it is necessary to decipher the systems-level mechanisms of PG to understand and further develop its therapeutic potential.

Network pharmacology is a novel approach for investigating the systems-level mechanisms of drugs [24]. It integrates multiple sources of information and adopts computational methods such as bioinformatics and network analysis, as well as experimental approaches. Recently, network pharmacological approaches were employed to investigate the systems-level mechanisms of herbs or herbal formulae, highlighting the potential of traditional herbal medicine in “multi-compound, multi-target” therapeutics [25,26,27,28,29].

**Figure 1:**
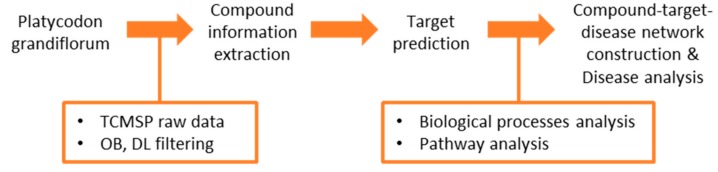
Framework of network pharmacological analysis of *Platycodon grandiflorum* (PG); TCMSP: Traditional Chinese Medicine Systems Pharmacology database; OB: oral bioavailability; DL: drug-likeness.

So far, there are several studies reviewing the therapeutic mechanisms of PG systemically based on individual experimental results; however, there were no attempts to apply network pharmacological analysis to decipher the systems-level mechanisms of PG. In this review, we attempt to provide comprehensive insight into the systems-level mechanisms of PG by adopting network pharmacological analysis. Firstly, we briefly introduce the chemical constituents that have a high possibility of being active compounds. Next, we constructed a compound–target–disease network using compound–target interaction data from the Traditional Chinese Medicine Systems Pharmacology database (TCMSP, http://lsp.nwu.edu.cn/tcmsp.php) [30]. In order to review the major targets of PG, the Uniprot database (https://www.uniprot.org/) was employed, and, to survey the pathways of selected targets, the Protein Analysis Through Evolutionary Relationships (PANTHER, http://www.pantherdb.org/) [31,32] classification system, Enrichr method (http://amp.pharm.mssm.edu/Enrichr/) [33,34], and clustergram method were applied [35]. Finally, absolute and relative degree matrices were constructed from a network of PG to investigate related diseases (Figure 1).

## 2. Compound Analysis

**Figure 2:**
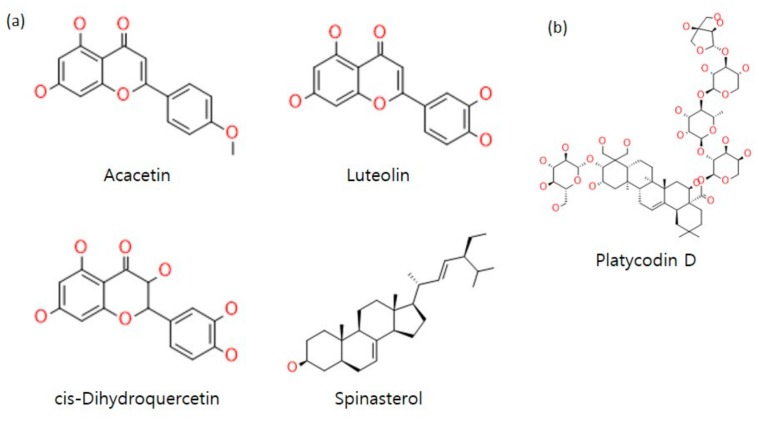
Chemical structures of PG compounds. (**a**) Four components of PG were selected from the TCMSP database with threshold values of 30 and 0.18 for OB and DL, respectively. (**b**) Platycodin D is a major active component of PG.

Among the compounds contained in the herb, not all compounds have drug characteristics. To search for compounds that have potential as a drug, we applied oral bioavailability (OB) and drug-likeness (DL) data to the compound the filtering process. [36]. OB is calculated based on permeability (P)-glycoprotein and cytochrome P450, which affect drug absorption and metabolism [37]. Meanwhile, DL is derived using Lipinski’s rule of five and Tanimoto coefficients [38]. To extract candidate compounds from PG, the thresholds of OB and DL were set to ≥30 (OB) and ≥0.18 (DL) and applied for filtering. Compound information was extracted from the TCMSP database [30]. The candidate compounds turned out to be as follows: acacetin, luteolin, *cis*-dihydroquercetin, spinasterol, robinin, 2-*O*-methyl-3-*O*-β-d-glucopyranosyl platycogenate, and dimethyl 2-*O*-methyl-3-*O*-α-d-glucopyranosyl platycogenate A. Among them, compounds with no interacting target were excluded, resulting in three flavonoids (acacetin, luteolin, *cis*-dihydroquercetin) and one steroid (spinasterol) (Figure 2a).

Acacetin was reported to inhibit the proliferation of cancer cells by blocking cell-cycle progression and inducing apoptosis. For example, it was demonstrated to obstruct the proliferation of cancer cells from liver, lung, prostate, and breast tumors [39,40,41,42]. Dihydroquercetin was shown to have neuroprotective and hepatoprotective activity through antioxidant effects [43,44]. Luteolin was found to promote antioxidant activity [45,46], lipolysis [47], and anti-angiogenic activity [48]. Spinasterol was investigated for anti-carcinogenic [49], anti-tumor [50], and anti-nociceptive effects [51].

It is worthy of note that there can be other potential therapeutic compounds of PG in addition to the compounds we suggested based on the TCMSP database and filtering process. For example, platycodin D is the most frequently reported active compound among PG compounds [52,53] although it showed OB and DL values below the threshold (7.60 and 0.01, respectively) (Figure 2b). Platycodin D was reported to have various pharmacological effects, such as inducing apoptosis [9,10,11,12,13], as well as anti-obesity [14,15] and anti-inflammatory [16,17,18] properties, increasing airway mucin release [19,20], and protection against hepatotoxicity [21,22].

## 3. Construction of PG Compound–Target–Disease (CTD) Network

In order to predict the systemic therapeutic effects of PG, we constructed a CTD network comprising three types of nodes (compounds, targets, and diseases) and two types of edges (between compounds and targets, and between targets and diseases; CT and TD interactions, respectively). CT and TD interaction information was extracted from TCMSP. CT interaction data include not only experimentally validated interactions, but also predicted interactions based on machine learning (ML) methods such as support vector machines and random forest algorithms. The performance of this ML-based method was proven to be reliable [54].

**Figure 3:**
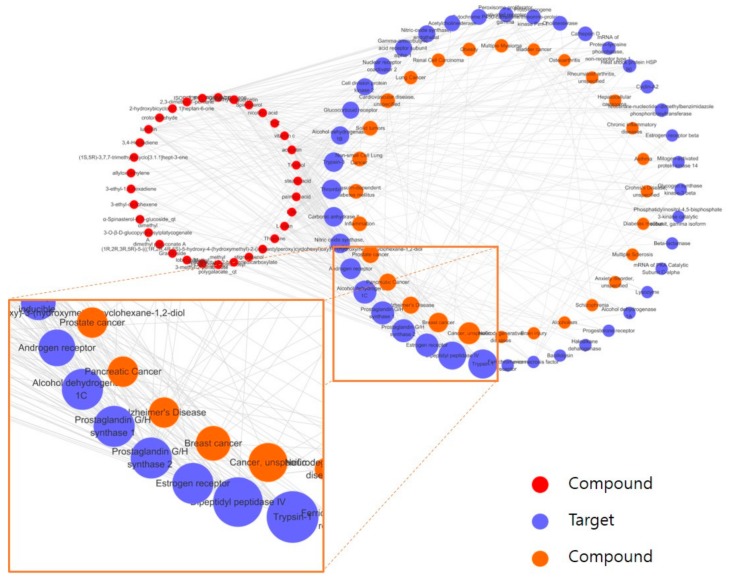
Compound–target–disease (CTD) network of PG. The red circles represent compounds, the purple circles represent targets, and the orange circles represent diseases. The compounds, targets, and diseases of each network are sorted in descending order from the bottom of the figure in a circular layout, on the basis of the number of degrees. The size of the target and disease nodes reflects the number of degrees. The node was removed when the number of degrees of the target and disease nodes was <3. The orange box represents the top five targets and diseases in terms of degrees.

The degree of each node was defined by the number of connections that the node has. Specifically, target degree was defined as the number of connections each target has to compounds, and disease degree was defined as the number of connections each disease has to targets. To explore as many potential targets and diseases of PG as possible, the thresholds of OB and DL were set to 0. Instead, we only included targets and diseases with more than three degrees in further analysis. Cytoscape 3.6.1 (http://www.cytoscape.org/) [55] was used to visualize the constructed network (Figure 3).

The CTD network constructed for PG contained 33 compounds, 40 targets, and 28 diseases. Five targets with the most degrees among the 40 targets were as follows: trypsin-1, dipeptidyl peptidase IV, estrogen receptor, prostaglandin G/H synthase 2, and prostaglandin G/H synthase 1; the top five diseases that were most relevant to PG were as follows: unspecific cancer, breast cancer, pancreatic cancer, Alzheimer’s disease, and prostate cancer.

## 4. Target Analysis

Since presenting only the name of the target has limitations in providing an understanding of the therapeutic effects of PG, we queried the top 10 biological functions of each target in Uniprot, a target annotation information database. Since many of the CT interactions in the network were based on the results of ML prediction without experimental validation, there is the possibility of spurious interactions in the network. Therefore, we mainly focused on targets with high numbers of degrees (top 10 targets) for target analysis. Among many target annotations in Uniprot, to find the pharmacological functions of PG, 12 pharmacological activities of PG were pre-selected based on two review papers [56,57], i.e., apophlegmatic and antitussive, immune, anti-inflammatory, anti-oxidant, anti-tumor and anti-cancer, anti-diabetic, anti-obesity, anti-allergic, anti-microbial, cardiovascular, hepatoprotective, and neuroprotective activities. The pharmacological activities of each target were retrieved from the annotation information of Uniprot and matched with the pharmacological activities of PG (Table 1).

Among the top 10 targets, *DPP4* is known as a gene involved in T-cell immune activation, and has a biological process of insulin secretion, as well as locomotor and psychomotor behavior. *ERS1* and *AR* are involved in gene expression control, which affect cell proliferation and differentiation of the target tissue. In particular, *ERS1* is known to act on cancer, tumor, and inflammation by controlling phosphatidylinositol 3-kinase (PI3K)/Protein Kinase B (Akt) signaling associated with cell abnormal proliferation [58], and *AR* is known to be associated with prostate cancer [59]. *PTGS2*, better known as *COX2*, is involved in prostanoid synthesis. This gene was reported to cause inflammatory responses and phenotypic changes, resistance to apoptosis, tumor angiogenesis, and cancer [60,61,62]. *NOS2* is a gene that produces nitric oxides (NOs), mediating tumoricidal and bactericidal actions in macrophages [63]. This gene is also closely related to the inflammatory response because it is involved in prostaglandin secretion [62]. *CA2* is known as the target of breast cancer and glaucoma treatment drugs [64]. *CA2* is also related to osteopetrosis because it plays an important role in bone resorption and osteoclast differentiation [65].

## 5. Pathway Analysis

Next, we performed pathway analysis using predicted targets of PG. Pathway analysis aims to provide insight into the biological processes involved in the predicted targets. Firstly, to capture the related biological functions of PG, every target of the CT network was assigned to biological processes using the “PANTHER GO-Slim Biological Process” feature of the PANTHER database [31].

**Figure 4:**
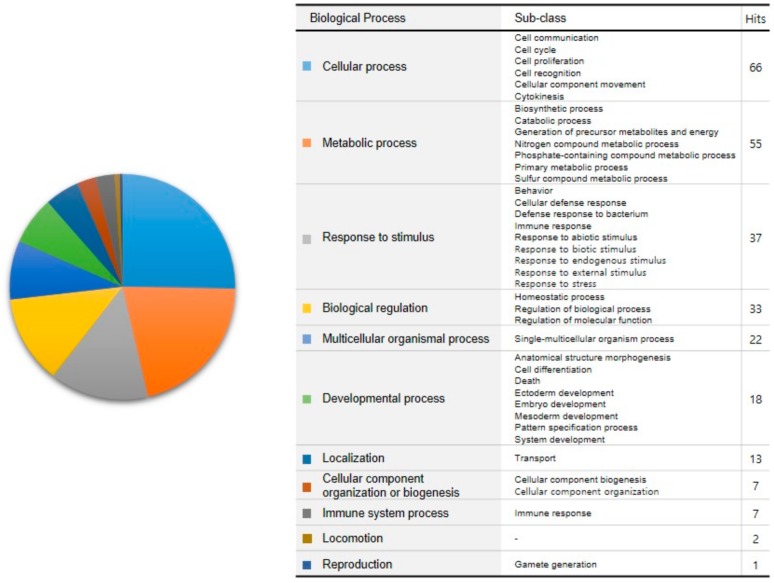
Biological processes related to targets of PG. Targets were assigned to biological processes using “Panther GO-slim Biological Process”. Hits mean the number of assigned targets to the corresponding biological processes. The proportion of each biological process is color-coded in the pie chart.

As a result, 112 targets were assigned to 261 biological processes. Biological processes were classified into 11 categories as follows: cellular process, metabolic process, response to stimulus, biological regulation, multicellular organismal process, developmental process, localization, cellular component organization or biogenesis, immune system process, locomotion, and reproduction (Figure 4)

**Figure 5:**
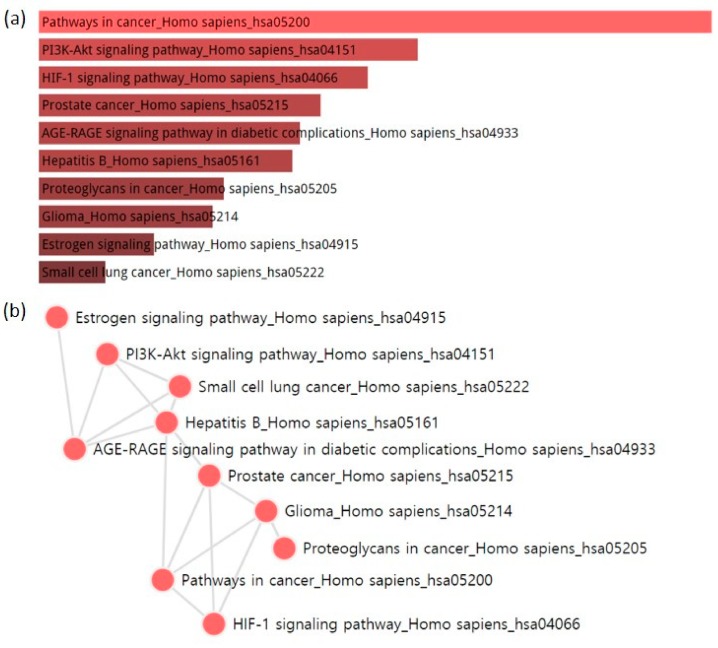
Pathway analysis using the Kyoto Encyclopedia of Genes and Genomes (KEGG) 2016 library. Analysis was performed on the “Enrichr” platform (http://amp.pharm.mssm.edu/Enrichr/). The 112 targets of PG were used to obtain the results. (**a**) The top 10 enriched pathway terms are displayed in a bar graph. They are ranked by a combined score calculated by *p*-value and *z*-score. The length of the bar and the brightness of its color represent the significance of the specific pathway. (**b**) The top 10 enriched pathway terms are displayed as a network. Each node represents a pathway, and each edge represents the gene content similarity among the pathways.

We also applied gene set enrichment analysis (GSEA) [66] to investigate target-related pathways. The pathway information of the Kyoto Encyclopedia of Genes and Genomes (KEGG) 2016 database (https://www.kegg.jp/) [67] was used for the enrichment analysis. The top 10 enriched terms were ranked in descending order as follows: pathways in cancer, PI3K/Akt signaling pathway, hypoxia-inducible factor 1 (HIF-1) signaling pathway, prostate cancer, advanced glycation end products (AGE)/receptor for advanced glycation end products (RAGE) signaling pathway in diabetic complications, hepatitis B, proteoglycans in cancer, glioma, estrogen signaling pathway, and small-cell lung cancer. These enriched terms were visualized as a bar graph using a combined score and as a network based on the gene content similarity among the enriched terms (Figure 5) [33,34]. The network of pathways provides information on how the diverse pathways are related in terms of the target genes of PG. We found that PG mainly acts on pathways related to cancers, and non-cancer pathways such as hepatitis B, AGE/RAGE, and glioma, which share target genes in common with various cancer-related pathways.

**Figure 6:**
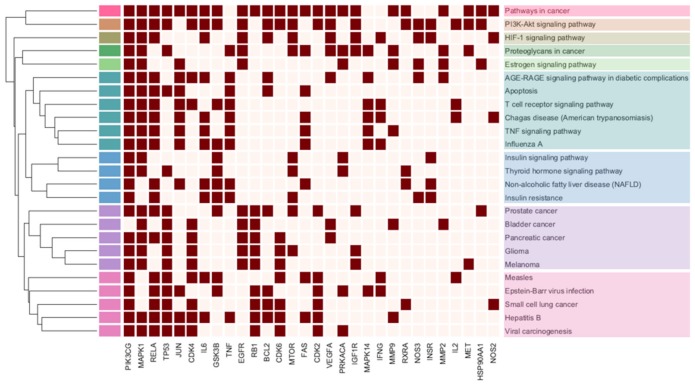
Clustergram of pathways. Rows and columns represent the pathways and input genes, respectively. The enriched terms in the rows of the heat map are clustered by the similarity of the gene contents. The input genes in the column are sorted in descending order of the sum of the columns from left to right. The colors of the boxes represent each cluster.

To show how the pathways share target genes in common in more detail, we constructed a clustergram of pathways and genes. Only pathways with combined scores greater than 20 were included in the clustergram (Figure 6). To define clusters, we cut the dendrogram at the fifth level, resulting in nine clusters (Table 2). We found that many of the clusters were cancer pathways or closely related to cancers (clusters 1, 2, 3, 4, 8, and 9). These findings are consistent with many previous studies of PG. For example, PG was reported to inhibit proliferation of HT-29 colon cancer cells by inducing apoptosis via both caspase-dependent and -independent pathways [5]. Also, Shin et al. reported the effects of platycodin D on the production of reactive oxygen species (ROS) and showed the association of these effects with apoptotic tumor cell death [68]. PI3K-Akt signaling pathway which stimulates cell growth and cell cycle progression, is closely related to oncogenesis, and has been reported as a major cancer control pathway of PG [69,70]. Also, several studies have reported that PG stimulates NO and TNF-α release and is able to upregulate iNOS and TNF-α expression for anti-tumor activity [71,72].

## 6. Disease Analysis

**Figure 7:**
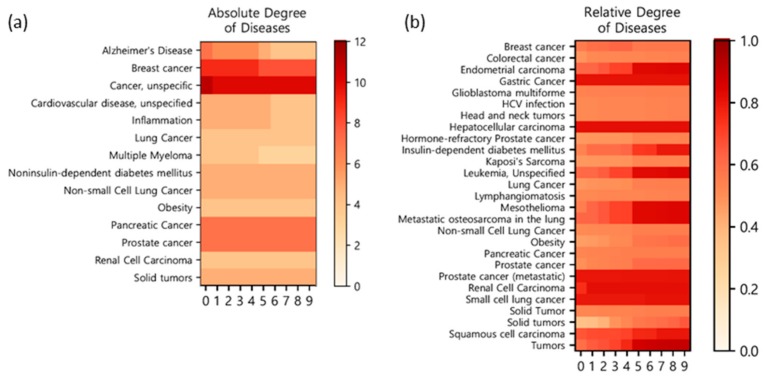
Degree matrices of the related diseases. Each row represents the major diseases of the CTD network, and each column represents the 10 thresholds of OB and DL. (**a**) The matrix shows the absolute degree of each disease. Only diseases with an average degree of 3 or higher are displayed. The color bar indicates the absolute degree of diseases. (**b**) The matrix represents the relative degree. Only the top 50% of diseases indicated by total herbs’ degrees are selected. The color bar represents the relative degree of diseases.

Finally, we analyzed the therapeutic effects of PG on diseases. Potential target diseases of PG were analyzed based on the target–disease information from TCMSP, which extracted information from PharmGKB (https://www.pharmgkb.org/) [73] and the Therapeutic Targets Database (http://bidd.nus.edu.sg/BIDD-Databases/TTD/TTD.asp) [74]. At first, disease degrees were calculated for all diseases in TCMSP by counting the number of interactions with targets in the constructed CTD network of PG (Figure 7a and Table 3). Since the results are affected by the selection of thresholds for OB and DL, the degrees of diseases were calculated across a wide range of thresholds, resulting in a degree matrix of diseases. In the matrix, only diseases whose average disease degree was >3 were displayed. Unspecific cancer shows the highest degree, followed by cancer-related diseases such as breast cancer, pancreatic cancer, and prostate cancer. Among non-cancer diseases, Alzheimer’s disease shows the highest degree.

The high proportion of cancer diseases in our results raised concerns that the results could be biased to specific diseases that have many related genes in the database (target genes are not evenly distributed for diseases). To avoid this bias, we calculated the relative degrees by dividing each degree by the maximum degree of the corresponding disease. The relative degree of a disease shows a comparative advantage of PG for various diseases by controlling for the frequency of the disease in the database (Figure 7b and Table 4). The results of the relative degree analysis were not identical with those of the absolute degree analysis, but the overall trend of a high proportion of cancer diseases was found again. Major diseases targeted by PG according to relative degrees are as follows: hepatocellular carcinoma, gastric cancer, prostate cancer, small-cell lung cancer, and renal cell carcinoma. Our network pharmacological analysis of target diseases of PG concurs with many previous studies on PG. The anti-cancer effect of PG was actively verified on various cancers such as hepatocellular carcinoma [11,12], lung cancer [4,10], breast cancer [13,75], colon cancer [5], and leukemia [68]. In addition, several studies described the beneficial effects of PG on various diseases such as anti-obesity [76,77] and neuroprotective [78] effects, and immune system activation [79,80].

## 7. Concluding Remarks and Future Directions

PG contains various ingredients, as well as platycosides (e.g., platycodin D), and these components could interact with multiple targets and pathways simultaneously in a complex manner to exert PG’s therapeutic effects. However, it is a challenging task to understand the complex mechanisms of action of PG at a systems level via conventional approaches based on reductive analysis. In the present study, we attempted to review the systems-level mechanisms of PG by applying network pharmacological methods, such as CTD network construction, target analysis, pathway analysis, and disease analysis using bioinformatics tools and databases. Our analysis revealed candidate targets of PG and target-related pathways which take the simultaneous actions of multiple compounds on multiple targets into account. We can also suggest potential target diseases of PG from this analysis, providing insight into PG’s therapeutic potential. We employed various analytical approaches to give reliable information at multiple levels and showed consistent results throughout the analysis. Furthermore, we tried avoiding publication bias that can occur when using bioinformatics databases in disease analysis, by considering the relative degree of diseases [25].

Although our network pharmacology-based review of systems-level mechanisms of PG is encouraging, a limitation should also be noted. Currently, there are several different approaches to each network pharmacological analysis step, such as the predictions of OB, DL, and drug–target interactions [36], and there is no consensus about which approaches are more appropriate for understanding the systems-level mechanisms of herbs with multiple components. Since the results of analysis are dependent on adopted methodologies, future studies are needed to optimize each step of the analysis by combining experimental validation data. It will also be necessary to incorporate multi-scale models of diseases and drugs based on systems-level experiments such as gene expression profiling, because they can provide more downstream results of complex interactions between multiple target genes [81,82]. In spite of this limitation, however, we found that our prediction-based results were generally consistent with previous research on pathways and diseases treated with PG extracts. Furthermore, we can suggest more comprehensive mechanisms of therapeutic effects of PG in terms of target proteins, pathways, and diseases than manual reviews of the literature. We expect that the review of systems-level mechanisms of herbs via network pharmacology will be a valuable approach for understanding and developing the therapeutic potential of herbs.

## Figures and Tables

**Table 1:** Top 10 targets in the compound–target (CT) network and pharmacological activity of each target.

Gene Name	Protein Name	Target Degrees	Pharmacological Activities in Uniprot
*PRSS1*	Trypsin-1	17	-
*DPP4*	Dipeptidyl peptidase IV	16	Immune activitiesAnti-diabetic activitiesAnti-microbial activities
*ESR1*	Estrogen receptor	12	Anti-inflammatory activitiesAnti-cancer activitiesAnti-tumor activities
*PTGS2*	Prostaglandin G/H synthase 2	12	Anti-inflammatory activitiesAnti-cancer activitiesAnti-tumor activities
*PTGS1*	Prostaglandin G/H synthase 1	12	Anti-inflammatory activities
*ADH1C*	Alcohol dehydrogenase 1C	12	-
*AR*	Androgen receptor	10	Anti-cancer activities
*NOS2*	Nitric oxide synthase, inducible	9	Anti-tumor activitiesAnti-microbial activitiesAnti-inflammatory activities
*CA2*	Carbonic anhydrase II	9	Anti-cancer activities
*F2*	Prothrombin	9	Cardiovascular activities

**Table 2:** Cluster of the enriched pathway. PI3K—phosphatidylinositol 3-kinase; HIF-1—hypoxia-inducible factor 1; AGE—advanced glycation end products; RAGE—receptor for AGE; TNF—tumor necrosis factor.

Cluster No.	Enriched Pathway
Cluster 1	Pathways in cancer
Cluster 2	PI3K signaling pathway
Cluster 3	HIF-1 signaling pathway
Cluster 4	Proteoglycans in cancer
Cluster 5	Estrogen signaling pathway
Cluster 6	AGE/RAGE signaling pathway in diabetic complications, apoptosis, T-cell receptor signaling pathway, Chagas disease (American trypanosomiasis), TNF signaling pathway, influenza A
Cluster 7	Insulin signaling pathway, thyroid hormone signaling pathway, non-alcoholic fatty liver disease (NAFLD), insulin resistance
Cluster 8	Prostate cancer, bladder cancer, pancreatic cancer, glioma, melanoma
Cluster 9	Measles, Epstein–Barr virus infection, small-cell lung cancer, hepatitis B, viral carcinogenesis

**Table 3:** The potential target diseases based on the absolute degree matrix of *Platycodon grandiflorum*.

Disease Name	Degree (From the 1st Level Threshold)	Disease Name	Degree (From the 1st Level Threshold)
Cancer, unspecific	11	Alcoholism	3
Breast cancer	9	Bladder cancer	3
Pancreatic cancer	7	Neurodegenerative diseases	3
Alzheimer’s disease	7	Brain injury	3
Prostate cancer	7	Schizophrenia	3
Inflammation	5	Hepatocellular carcinoma	3
Cardiovascular disease, unspecified	5	Multiple sclerosis	3
Non-small-cell lung cancer	5	Chronic inflammatory diseases	3
Non-insulin-dependent diabetes mellitus	5	Diabetes mellitus	3
Solid tumors	5	Osteoarthritis	3
Renal cell carcinoma	4	Anxiety disorder, unspecified	3
Obesity	4	Rheumatoid arthritis, unspecified	3
Multiple myeloma	4	Crohn’s disease, unspecified	3
Lung cancer	4	Asthma	3

**Table 4:** The potential target diseases based on the relative degree matrix of *Platycodon grandiflorum*. HCV—hepatitis C virus.

Disease Name	Relative Degree (From the 1st Level Threshold)	Disease Name	Relative Degree (From the 1st Level Threshold)
Hepatocellular carcinoma	0.82	Pancreatic cancer	0.53
Gastric cancer	0.81	Solid tumor	0.53
Prostate cancer (metastatic)	0.80	HCV infection	0.52
Small-cell lung cancer	0.79	Head and neck tumors	0.52
Renal cell carcinoma	0.75	Lymphangiomatosis	0.52
Squamous cell carcinoma	0.66	Non-small cell lung cancer	0.51
Endometrial carcinoma	0.62	Prostate cancer	0.49
Metastatic osteosarcoma in the lung	0.62	Colorectal cancer	0.48
Leukemia, unspecified	0.61	Hormone-refractory prostate cancer	0.47
Tumors	0.59	Kaposi’s sarcoma	0.47
Breast cancer	0.56	Lung cancer	0.47
Mesothelioma	0.55	Obesity	0.45
Glioblastoma multiforme	0.54	Solid tumors	0.34
Insulin-dependent diabetes mellitus	0.53		
